# Books and Authors

**Published:** 1909-11

**Authors:** 


### BOOKS AND AUTHORS.

---

**The Principles of Pharmacy.** By **Henry V. Arny**, Ph.G., Ph.D., Profeessor of Pharmacy at the Cleveland School of Pharmacy, Pharmacy Department of Western Reserve University. Octavo of 1,175 pages, with 246 illustrations, mostly original. Philadelphia and London: W. B. Saunders Company, 1909. (Cloth, $5.00 net; half-morocco, $6.50 net.)

A new book by a new author enters the field of pharmacy, and it is most welcome. Professor Arny divides his subject into seven parts. The first part deals with pharmaceutic processes, a striking feature being the clear discussion of the arithmetic of pharmacy. The second part deals with galenic preparations of the pharmacopeia and those unofficial preparations of proved value. The third part deals with the inorganic chemicals used in pharmacy. The fourth part discusses the organic chemicals used in pharmacy. The fifth part presents a systematic grouping of all the chemical tests of the pharmacopeia. The sixth part discusses the prescription from the time it is written until it is dispensed. The seventh part is devoted to laboratory work. The methods of Arny are novel,—his methods of teaching we mean,—and he arranges his book in a novel manner. He exhibits the practical in his instructions which, after all, is the better way, the majority being in search of that which will prove most valuable for sick people. Professor Arny has chosen well in the title of his book,—The Principles of Pharmacy.

In giving the working processes of the pharmacal laboratory, which we think the proper designation for the prescription department of the drug store, the author is clear, concise, and scientific. He wastes no words but goes at his subject as though he enjoyed it. In describing pharmaceutical, he is equally interesting. Here is a sample of expression that cannot be excelled. "Waters are *aqueous solutions of volatile substances,* not like spirits which are alcoholic solutions of volatile substances; not like solutions, which are aqueous solutions of non-volatile substances."

The author devotes two chapters to the prescription, which are the best commentaries we have seen on this topic. Every young physician,—every beginner we mean,—should read what Arny has to say on this topic. His facsimiles of dangerous prescriptions are examples of carelessness of which no physician should be guilty. Abbreviations especially are to be used with caution or, better, not at all.

The entire book is to be commended for its scientific treatment of the subject with which it deals, for the clear and expressive English in which its several parts are clothed, and as being a distinct addition to the literature of pharmacy.

---

**American Practice of Surgery.** A complete System of the Science and Art of Surgery by representative surgeons of the United States and Canada. Edited by **Joseph D. Bryant,** M.D., and **Albert H. Buck,** M.D., New York. Complete in eight volumes. Vol. VI. Imperial octavo, pp. 916. Profusely illustrated. New York: William Wood & Company. 1909. (Price, cloth, $7.00.)

Regional surgery is continued in this volume the titles being: Prosthesis in its relation to surgery of the face, mouth, jaws, and nasal and laryngeal cavities, by Charles R. Turner, of Philadelphia; Surgical diseases and wounds of the nasal cavities and accessory sinuses, by Harris Peyton Mosher, of Boston; Surgical diseases and wounds of the mouth, tongue and salivary glands, by George Armstrong, of Montreal; Surgical diseases and wounds of the neck, by John M. Elder, of Montreal; Surgical diseases and wounds of the thyroid and thymus, by Francis J.

Shepherd, of Montreal; Surgery of the thorax and spinal column, by Norman B. Carson, of St. Louis; Surgical diseases and wounds of the female breast, by Harvey G. Mudd, of St. Louis; Surgical diseases and wounds of the external genitals and vagina of the female, by William P. Graves, of Boston; Surgical diseases and wounds of the male genital organs, by Franklin G. Balch, of Boston; Chancroid, by Hugh Cabot, of Boston; Gonorrheal urethritis, by Hugh Cabot, of Boston, and Surgical diseases and wounds of the jaws, by Joseph C. Bloodgood, of Baltimore.

None of the previous volumes has exceeded this one in interest. The section on the diseases and wounds of the nasal cavities and accessory sinuses by Mosher, is unusually well presented and will attract the attention of specialists, as well as general surgeons. This great work is nearing completion, but two more volumes being promised. It will constitute when finished, a lasting monument to the achievements of American surgeons who have participated in its creation.

---

**Progressive Medicine,** Vol. III, September, 1909. A Quarterly Digest of Advances, Discoveries and Improvements in the Medical and Surgical Sciences. Edited by **Hobart Amory Hare**, M.D., Professor of Therapeutics and Materia Medica in the Jefferson Medical College of Philadelphia. Octavo, 336 pages, with 37 engravings. Lea & Febiger, Publishers, Philadelphia and New York. (Per annum, in four cloth-bound volumes, $9.00; in paper binding, $6.00, carriage paid to any address.)

This number of the quarterly digest presents four topics by four men of experience. The first section deals with diseases of the thorax and is prepared by Professor Ewart, of London. He includes diseases of the bloodvessels in his summary. Dermatology and syphilis constitutes the second section, which is arranged by William S. Gottheil, of New York. He includes in his article Dr. Grover W. Wende's case of erythema figurate, which is illustrated. The third section, by Edward P. Davis, of Philadelphia, is devoted to obstetrics, in which are considered pregnancy and its complications, labor, obstetric surgery, the puerperal period, and the newborn. The fourth and final section presents diseases of the nervous system, and is prepared by William G. Spiller, of Philadelphia. This is a number of exceptional interest.

---

**A Theory Regarding the Origin of Cancer.** By **C. E. Green**. Second edition. Edinburgh and London: William Green & Sons. 1909.

A primer that offers a theory regarding cancer at the present time is, indeed, a bold little book. The finger and toe diseases of plants is made to do duty here and the author offers an ingenious comparison between it and the fungoid organism fostered by manures that have been dissolved in sulphuric acid! However, it is better to read this book than to undertake a criticism of its theory. Let the "cancer specialist" look to his laurels!

---

**The Practical Medicine Series.** Comprising ten volumes on the year's progress in medicine and surgery, under the general charge of **Gustavus P. Head,** M.D., Professor of Laryngology and Rhinology at the Chicago Postgraduate Medical School. Vol. IV., Gynecology. Edited by **Emilius C. Dudley,** A.M., M.D., Professor of Gynecology Northwestern University Medical School and **C. von Bachellé,** M.S., M.D., Assistant Professor of Obstetrics Chicago Polyclinic and College of Physicians and Surgeons. Series, 1909. Chicago: The Year Book Publishers. (Price, $1.25.)

This compilation by E. C. Dudley and C. von Bachellé consists of six parts, the first dealing with general principles; the second with infections and allied disorders; the third with malformations and tumors; the fourth with traumatism; the fifth with displacements; and the sixth with disorders of menstruation and sterility. In the second part reference is made to the paper of Dr. C. M. Rees, of Charleston, S. C., on arteriosclerosis of the uterus, read at Baltimore last year, before the American Association of Obstetricians and Gynecologists. Hemorrhage in many of these cases is such as to endanger life in which condition the only remedy is hysterectomy. Considerable space is given to perineorrhaphy, the newly devised operations being several in number, some of which are illustrated. At most they are, in general, modifications of methods already in vogue. The little volume is full of excellent hints on gynecological procedures.

---

**Lectures on Hysteria and Allied Vasomotor Conditions.** By **Thomas Dixon Saville,** M.D., Lond., Physician to the West End Hospital for Diseases of the Nervous System, and to the Saint John's Hospital for Diseases of the Skin, Leicester Square, London. New York: William Wood & Co. London: Henry J. Glaisher. 1909. (Price, $2.50.)

The lectures delivered by the author at the West End (London) Hospital for diseases of the nervous system, on hysteria and allied vasomotor conditions, are here presented in book form at the request of several friends. Saville has distinguished himself by presenting to an American audience his work on neurasthenia, also in the form of lectures delivered at the same hospital. He says the essential cause of this disease is an unborn predisposition to develop hysterical manifestations, which exists throughout life, this constituting the hysterical diathesis or temperament. Professor Saville is interesting as well as clear in his description of the various manifestations of this strange malady, and has presented a valuable contribution to the study of the topic. His book will claim the attention not only of general practitioners but specialists as well.

**Exercise in Education and Medicine.** By **R. Tait McKenzie**, A.B., M.D., Professor of Physical Education, and Director of the Department, University of Pennsylvania. Octavo of 406 pages, with 346 illustrations. Philadelphia and London: W. B. Saunders Company, 1909. (Cloth, $3.50 net; half-morocco, $5.00 net.)

Exercise is an important, even a large part of one's school and college training nowadays. Sometimes it is overdone and leads to breakdown prematurely, when excessive in weak constitutions. The object of this book is to place in the hands of students and teachers of physical training, to teachers of youth, and students and practitioners of medicine, a comprehensive view of the space to be given exercise in the scheme of education and as a method of the treatment of abnormal conditions. Education without health, or at the expense of health is of little or no value. Every young person should understand his own limitations regarding exercise, and especially such violent exercise as pertain to football and race rowing.

It is the province of this work to instruct in such exercise as will promote good health and preserve the forces nature provides for the individual with which to enjoy life and attain longevity. Almost every form of gymnastic exercise is here described and illustrated; indeed, the illustrations speak in clear and emphatic tones of the value of the text. Many of the movements are useful in the correction of deformities, such as lateral curvature of the spine, while others serve to develop muscular strength in those preternaturally weak.

Of all the books relating to physical exercise we have seen this is one of the very best. It should be in the hands of teachers of physical culture, as well as physicians, while the laity could learn much from its pages were they carefully studied.

---

**Clinical Treatises on the Symptomatology and Diagnosis of Disorders of Respiration and Circulation.** By **Professor Edmund von Neusser**, M.D., Professor of the Second Medical Clinic, Vienna; Associate Editor of Nothnagel's Practice of Medicine. Authorised translation by **Andrew MacFarlane**, M.D., Professor of Medical Jurisprudence and Physical Diagnosis at the Albany Medical College, etc. Part III, Angina Pectoris. New York: E. B. Treat & Company. 1909. (Price, $1.00.)

Angina pectoris is a symptom not a disease; nevertheless, it is a symptom of such magnitude, such urgency as to assume the dignity, the clinical importance of a disease. Usually a disease of advanced life, it occasionally attacks younger persons, hence the clinician must be on his guard to recognise its classic symptoms at any time of life. When accompanied by a weak or degenerated heart and when the coronary artery is narrowed the prognosis is doubtful; indeed, even the initial attack may prove fatal. On the other hand, with a comparatively sound heart and undamaged coronaries much may be expected from a well directed therapy. This monograph contains much of value and will serve to give useful hints on pathology and treatment.

**Dorland's American Illustrated Medical Dictionary.** Fifth edition. By **W. A. Newman Dorland,** M.D., large octavo of 876 pages, with 2,000 new terms. Philadelphia and London. W. B. Saunders Company, 1909. (Flexible leather, $4.50 net; indexed, $5.00.)

Five editions of a medical dictionary in nine years are a good many, and testify to the activity of the author in keeping his work well in the forefront of progress. Since the previous edition, the author has searched the medical literature for new words and has incorporated in this book more than 2,000 new terms, the majority of which he says appears for the first time in any dictionary. He has also revised the entire book, rewriting and otherwise improving many of the definitions. Much new matter has been added relating to the terminology of parasites, particularly of the protozoa; also the B. N. A. anatomical terminology has been included, and the latest classification of the proteins has been inserted. The illustrations throughout the book, some in colors, are of exceptional quality, artistic, helpful in making the text clear, and deserving of praise.

---

**Clinical Treatises on the Pathology and Therapy of Disorders of Metabolism and Nutrition.** Part VIII. Gout, by Professor **Dr. H. Strauss,** Professor of the Third Clinic, Royal Charity Hospital, Berlin. Authorised American Edition, translated under the direction of **Nellis Barnes Foster,** M.D., Associate Physician, New York Hospital, etc., New York; E. B. Treat & Company. 1909. (Price, $1.00.)

Gout is a disease that commands the attention of the profession at the present day more than ever before. This essay gives a concise picture of modern conceptions of the nature of gout and points out the rational treatment of the disease. It is one thing to treat the attack,—the acute attack we should have said,—and another to deal with the diathesis. This author handles both with rare judgment. The monograph will claim the attention as it deserves the respect of the American profession.

---

**Bier's Hyperemic Treatment in Surgery,** Medicine and all the Specialties. By **Willy Meyer,** M.D., Professor of Surgery at the New York Post-Graduate Medical School and Hospital: and Professor **Dr. Victor Schmieden,** Assistant to Professor **Bier** at Berlin University, Germany. Second edition. Octavo of 280 pages, illustrated. Philadelphia and London: W. B. Saunders Company, 1909. (Cloth, $3.00 net.)

This book was printed first in March, 1908, and has been printed four times since that date. This revision is the fifth printing in say fifteen months. Evidently there is a demand for the work, as we intimated there might be in our notice of the first edition. Some revision has been made of the text, some additions have been made, and an index has been supplied of the world's literature relating to Bier's hyperemic treatment. The book still remains the authoritative exponent in America of this method of applying hyperemia to the treatment of disease

**Rational Immunisation in the Treatment of Pulmonary Tuberculosis and other diseases,** comprising a paper read before the Royal Society of Medicine, March, 1909. By **E. C. Hort,** B.A., B.Sc., M.R.C.S. New York: William Wood and Company. 1909.

The essays contained in this well printed little volume will prove of interest to all engaged in the study of immunisation. Mr. Hort has something to say and says it well, no matter whether all agree with him or not. A number of charts are inserted to give force to some of his clinical notes. Such monographs serve to give direction to the study of the complex topics dealt with and to steady opinions that may be liable to radical expression, if not held in check by a lever tempered with conservatism.

---

**The Practical Medicine Series.** Comprising ten volumes on the year's progress in medicine and surgery; under the general charge of **Gustavus P. Head,** M.D., Professor Laryngology and Rhinology at the Chicago Postgraduate Medical School. Vol. V. Obstetrics. Edited by **Joseph B. DeLee,** A.M., M.D., Professor of Obstetrics at the Northwestern University Medical School, with the Collaboration of **Herbert M. Stowe,** M.D. Series of 1909. Chicago: The Year Book Publishers. 1909.

In this volume the compiler presents the advances in obstetrics during the year 1908. While nothing startling is recorded in it, yet DeLee offers a book full of interest to the general practitioner who must of needs include obstetrics in his work. In part first pregnancy is dealt with, including its physiology, pathology, diagnosis, hemorrhages, toxemia, and presentations. In part second, devoted to the consideration of labor, are found anesthesia in labor, management of labor, pathology of labor, operative obstetrics, and puerperal sepsis. The puerperium constitutes the subject of the third part, which includes the management of the puerperium, injuries of the newborn, and the pathology of the newborn. The book is an excellent one for the general practitioner who wishes quick reference to recent literature.

---

**The Ophthalmic Year Book,** Volume VI. By **Edward Jackson,** A.M., M.D., Professor of Ophthalmology at the University of Colorado; **George de Schweinitz,** A.M., M.D., Professor of Ophthalmology at the University of Pennsylvania; and **Theodore B. Schmeideman,** A.M., M.D., Professor of Ophthalmology at the Philadelphia Polyclinic. Illustrated. The Herrick Book and Stationery Company, Denver. 1909.

In this book is gathered for reference the literature of ophthalmology for the previous year, that is to say, it gives in abstract the advances in ophthalmology for the year 1908. It presents the opposing views of different authors on subjects of importance and is of special value to those who would prepare society papers or yet more pretentious monograph, and even textbooks or manuals. It contains several illustrations of value, but these might be increased, it seems to us, with benefit to the book. The frontispiece is a portrait, bust size, of Herman Snellen, Sr., who died January 18, 1908. A number of biographical sketches have been inserted, including one of Snellen himself. At the end, just before the index, is a list of books, monographs, and journalistic articles published during 1908. which is very complete indeed. This is by far the best compilation of the literature of any specialty we have seen.

---

**Obstetrics.** A Manual for Students and Practitioners. By **David J. Evans,** M.D., Lecturer on Obstetrics in McGill University, Montreal. Second edition. 12mo, 440 pages, with 169 illustrations. Lea & Febiger, Philadelphia and New York, 1909. (Cloth, $2.25, net.)

Almost every teacher of obstetrics feels impelled to prepare either a handbook or a manual or, perhaps, a textbook on midwifery. This would seem to account for the fact that the literature relating to obstetrics has become somewhat overcrowded. This author, however, has a reason to speak because he understands his subject and has demonstrated the fact that he knows how to impart knowledge to his pupils; more than this, even, he has been successful in instructing that larger audience to which he speaks through the medium of his writings. The first edition of this book was exhausted promptly, though neither the author nor the publishers are kind enough to give us the date of its issue. We are of the opinion that each edition of a book should be dated. In after years it may be important to possess this information.

In this edition three sections,—those dealing with the implantation of the ovum, the development of the placenta, and toxemia, —have been rewritten. Others have been changed, either enlarged or decreased according to their importance, while the entire book has been revised. As at present constituted, it is one of the best manuals on obstetrics with which we are familiar.

---

**The Malarial Fevers, Hemoglobinuric Fever and the Blood Protozoa of Man.** By **Charles F. Craig,** M.D., Captain Medical Corps United States Army, Attending Surgeon New York. New York: William Wood & Company. 1909. (Price, $4.50.)

The writer of this book is recognised as being high authority on malarial fevers. He has published, heretofore, monographs, besides separate articles for Allbutt's system of medicine and Osler's Modern Medicine, as well as his own work on estivo-autumnal malarial fevers (1901), hence he has become quite well known to the profession as a writer on this subject. The present work he says is principally the result of his personal experience, obtained in Cuba and the Philippines and in the United States military hospitals, covering ten years of investigation.

The author has offered in this book the most pretentious treatise yet presented on this topic,—on the fevers of tropical and subtropical countries. Craig gives a terse, clear definition of "the malarial fevers," and says "malaria," though faulty, must be retained because it has become so firmly established in medical nomenclature—a statement with which we are in complete accord.

The author discusses the etiology of the malarial fevers in five chapters, which are models for their scientific treatment of the topic, and for their rhetorical finish. The mosquito, as we would expect, comes in for its full share of blame in the transmission of malaria; indeed, Craig asserts, that the bite of infected mosquitos is the only way as yet actually proven in which these fevers are naturally transmitted.

The entire treatise is of absorbing interest and should be studied by every medical officer, and every physician likely to do duty in tropical regions, or to practise in the tropics. It is a well printed book and is appropriately illustrated. At the end of each chapter, too, there is an exhaustive list on the literature of the topic dealt with.

---

**Report of the Commissioner of Education** for the year ended June 30, 1908. Vol. 2. Washington: Government Printing Office.

This volume concerns itself chiefly with statistics, including those pertaining to common schools, universities, colleges, technical schools, agricultural and mechanical colleges, professional schools, normal schools, secondary schools, manual and industrial training, commercial and business schools, training schools for nurses, schools for the colored race, reform schools and schools for the defective classes. The report on education in Alaska is then presented, the volume concluding with a summary of the statistical tables. The book is a fitting supplement to the preceding volume.

---

**Physiological and Medical Observations among the Indians of Southwestern United States and Northern Mexico.** By **Alex. Hrdlicka.** Washington: Government Printing Office. 1908.

This is a book of great interest to the student of anthropology and ethnology. It gives in narrative form the observations made by the author, extending over several years, in the course of six expeditions to the Indian tribes in the southwestern portion of the United States and northwestern Mexico. They chiefly relate to physiology and medicine, that is to say, they are chiefly physiological and medical observations. The climate, population, dwellings and social conditions are considered, and numerous illustrations indicate environment and methods and habits of the various tribes. Diseases to a certain extent are dealt with but only in a cursory way. The book, as we have said, is full of interest, and should prove a stimulus to further observations along the same lines.

**Tuberculosis, a Preventable and Curable Disease.** Modern methods for the solution of the tuberculosis problem, by **S. Adolphus Knopf,** M.D., Professor of Phthisiotherapy at the New York Post-graduate Medical School and Hospital. New York: Moffat, Yard & Company. 1909.

It will not be because of faulty or insufficient literature if tuberculosis is not stamped out or its spread controlled. For sometime past Knopf has been recognised as authority on tuberculosis, and we are glad to see him in print once more in such a substantial book. We think the title of this work well chosen. It catches the eye to affirm in bold type that tuberculosis is a preventable and a curable disease. It is not so very long ago, that this disease was considered neither preventable nor curable. At first, even the profession was startled by the announcement, then doubtful, and even denied the statement, while the laity was unconvinced for many years, and even yet but a small proportion accept the truth. Now, however, that an unrelenting campaign of information, of instruction, is waging throughout the civilised world converts appear daily in large numbers, and the hope is cherished that within a few years it will become an accepted fact among intelligent people, even among those who are unlettered, that tuberculosis is a preventable and a curable disease.

Such books as this will do much toward educating the people on the lines mapped out in the foregoing paragraph. The book begins by telling in its first chapter what a tuberculous patient should know of his disease; tells of the duties of the people, of the clergy, of physicians; tells how a sanatorium should be constructed for tuberculous patients, in short, gives the duties of everybody concerned, of methods and habits of life indoors and outdoors, and almost every other thing of importance to know, concerning the prevention and management of the disease.

---

**Myomata of the Uterus.** By **Howard A. Kelly,** Professor of Gynecology in the Johns Hopkins University. And **Thomas S. Cullen,** Associate Professor of Gynecology, Johns Hopkins University. Octavo, pp. 723. Illustrated. Philadelphia and London: W. B. Saunders Co. (Price, $7.50.)

This remarkable work represents an enormous amount of labor, covering fifteen years of time, and embracing the examination of 1,674 cases. During the period of time over which this investigation has extended, the operative procedures pertaining to uterine myomata have developed toward perfection in an amazing degree. Twenty years ago many cases were considered inoperable that now are undertaken with confidence. The surgical methods have marked an evolution, so to speak, from the strong rubber ligature placed temporarily around the cervix to control hemorrhage, along the period of the control of each of the principal vessels, to the bisection of the cervix, and now even to the transverse severance of the cervix when indicated. It may be affirmed as asserted by Cullen that a myomatous uterus that cannot be removed under one method or another, is a rarity, even a condition so extreme as to take rank nowadays as a curiosity.

Cullen adopts the term myoma as being simple and expressive, covering the tumors known as myomata, fibromata, fibromyomata, and fibroids, these all meaning precisely the same thing. He describes, clinically, almost every form of myoma possible to exist, and many of these he illustrates from the pathological specimens removed. Every variety of degenerative change is also described, some of which are illustrated, and all are dealt with in detail. The book is one of the most important contributions to the study of uterine myomata, yet presented to the professional world. It also will serve as a guide to surgical procedures undertaken for the cure of these tumors. These several methods are graphically illustrated in the concluding chapters of the book.

---

**Medical Jurisprudence, Forensic Medicine and Toxicology.** Prepared under the editorship of **R. A. Witthaus,** M.D., Professor of Chemistry, medical jurisprudence and toxicology Cornell University and **Tracy C. Becker,** A.B., LL.B., professor of general law and medical jurisprudence, University of Buffalo. Second edition. Volume III. New York: William Wood & Company. 1909. (Price, $6.00 a volume.)

This volume contains medico-legal relations of vision and audition and of injuries of the eye and ear, by J. H. Woodward; medico-legal relations of insurance, by A. L. Becker; medical aspects of insanity in its relations to medical jurisprudence, by Edward D. Fisher; mental unsoundness in its legal relations, by Tracy C. Becker and Charles A. Boston; care and custody of incompetent persons and their estates, by Goodwin Brown, revised by A. L. Becker; medico-legal aspect of marriage and divorce, by A. L. Becker; medico-legal relations of *x*-rays and skiagraphs, by Albert G. Geyser; medico-legal examination of blood and other stains and of the hair, by James Ewing.

These several sections present material full of interest to both physician and jurist, all being prepared by men of scientific attainments in medicine or of learning in the law. The section on the medico-legal relations of *x*-rays and skiagraphs deals with one of the newer questions of medical jurisprudence and possesses considerable interest to the amateur skiagraphist, who either burns himself or his patient, through his carelessness or want of skill.

The entire volume contains valuable instruction suited to both physician and lawyer who would acquaint himself with the importance of medico-legal topics; and especially the expert witness may find aid within its pages to help him over difficult places.

**Manual of the Diseases of the Eye.** By **Charles H. May**, M.D., Attending Ophthalmic Surgeon to the Mt. Sinai Hospital. Sixth edition. Illustrated. New York: William Wood & Company. 1909. (Price, $2.00, net.)

When first this manual appeared nine years ago, it took root in the favor of the profession, not only the ophthalmic profession but with the general practitioners who know something about the eye and its diseases. It has gone forward steadily from that time, increasing its popularity, having now reached its sixth edition. Every page in this edition has been examined by the author with care. Many alterations having been made, and the whole topic brought down to the immediate present. Some additions, too, have been made especially to such subjects as trans-illumination, the conjunctival tuberculin test, cerebral decompression, and the like. It easily takes its place among the foremost manuals on diseases of the eye.

